# Raman Tweezers Spectroscopy of Live, Single Red and White Blood Cells

**DOI:** 10.1371/journal.pone.0010427

**Published:** 2010-04-29

**Authors:** Aseefhali Bankapur, Elsa Zachariah, Santhosh Chidangil, Manna Valiathan, Deepak Mathur

**Affiliations:** 1 Centre for Atomic and Molecular Physics, Manipal University, Manipal, India; 2 Department of Pathology, Kasturba Medical College, Manipal, India; 3 Tata Institute of Fundamental Research, Mumbai, India; The University of Manchester, United Kingdom

## Abstract

An optical trap has been combined with a Raman spectrometer to make high-resolution measurements of Raman spectra of optically-immobilized, single, live red (RBC) and white blood cells (WBC) under physiological conditions. Tightly-focused, near infrared wavelength light (1064 nm) is utilized for trapping of single cells and 785 nm light is used for Raman excitation at low levels of incident power (few mW). Raman spectra of RBC recorded using this high-sensitivity, dual-wavelength apparatus has enabled identification of several additional lines; the hitherto-unreported lines originate purely from hemoglobin molecules. Raman spectra of single granulocytes and lymphocytes are interpreted on the basis of standard protein and nucleic acid vibrational spectroscopy data. The richness of the measured spectrum illustrates that Raman studies of *live* cells in suspension are more informative than conventional micro-Raman studies where the cells are chemically bound to a glass cover slip.

## Introduction

Optical tweezers are proving to be of widespread utility in contemporary research in a number of fields, particularly in the biomedical sciences [1], [2]. Optical tweezers use tightly-focused laser light to create a sharp intensity gradient over tiny spatial dimensions such that microscopic dielectric objects floating in liquid media may be trapped by the action of a gradient force [3]. Such trapped objects can then be subjected to spectroscopic investigation [4]–[22] in such manner that the deleterious effects on spectral quality of the inevitable Brownian motion are circumvented. By combining spectroscopic techniques with optical tweezers, the possibility opens of being able to extract precise information about biochemical changes at a single-cell level *under physiological conditions* and without the necessity of chemical fixing of cells. Cell immobilization by chemical or physical means alters the physiochemical microenvironment and may give rise to changes in electrochemical potentials across the cell membrane that, in turn affect cellular functions [5]. In order to facilitate spectroscopic studies of single living cells in a physiological medium, we have developed a high-resolution, dual-wavelength apparatus that combines optical trapping with Raman spectroscopy, utilizing near infrared wavelength light at 1064 nm for trapping and 785 nm light for Raman excitation at very low levels of incident power (<10 mW). We have utilized this apparatus to probe, in sensitive and high-resolution fashion, Raman features in red and white blood cells.

Among several spectroscopy techniques, Raman spectroscopy is a particularly potent tool to probe the biochemical composition of cells; it has proved to have the potential of being able to differentiate between, for example, normal and malignant cells [9]–[11]. In recent years, micro-Raman spectroscopy has developed into a powerful tool to spectroscopically probe single cells with high spatial resolution, requiring relatively simple sample preparation procedures [23]–[27]. Resonance Raman spectroscopy has also been utilized to probe DNA bases and aromatic amino acids in proteins, and the technique has been successfully applied to rapidly identify bacteria such as *E. coli, P. fluorescens, S. epidemis, B. subtilis* and *E. cloaca* [26], [27].

The relatively recent marriage between the techniques of optical trapping and Raman spectroscopy [4], giving rise to Raman Tweezers, has resulted in a number of “offspring” in the form of interesting and important applications in the biomedical sciences [4]–[22]. This marriage has opened new vistas for biomedical applications that require spectroscopic information with good spatial resolution and identification of intra-cellular components under physiological conditions. The early examples of Raman Tweezers utilized a single, low power laser beam, usually of 785 nm wavelength, that was used for both optical trapping and near-infrared Raman spectroscopy [4]. The advantages of using a dual wavelength set-up for confocal Raman spectroscopy were established by Petrov and coworkers [6]; one of the two laser beams was used for trapping micron-size cells and a second laser beam was used to carry out Raman excitation.

Applications of Raman Tweezers to studies of red blood cells (RBCs) have attracted considerable contemporary attention [16]–[20]. Raman spectra have been measured of red blood cells in their equilibrium geometry as well as when they are stretched; spectral changes have been interpreted in terms of mechanically induced effects and indications have been obtained that stretched RBCs have significantly enhanced oxygen concentration [16]. Raman Tweezers experiments have also been reported that seek to examine the oxygenation capability of β-thalassemic RBCs and to probe the response to photo-induced oxidative stress of diseased cells with respect to normal ones [17]. The effect of alcohol on single RBCs has been reported [18] wherein the vitality of cells was characterized by the porphyrin breathing mode in Raman spectra at 752 cm^−1^; the intensity of this band decreases with alcohol concentration [18].

We describe in the following details of a successful coupling in our laboratory of a Raman spectrometer with an optical trap that allows, without physical contact, simultaneous optical trapping, optical manipulation, and recording of Raman spectra of micron-sized objects floating in a medium. The trapping is accomplished using tightly-focused, 1064 nm wavelength laser light while Raman spectra are recorded by exciting the trapped particle by 785 nm laser light. We have recorded Raman spectra of single, living red and white blood cells under physiological conditions, achieving an enhancement of overall spectral quality that has enabled identification of several additional lines in the Raman spectra of trapped cells.

## Methods

### Ethics Statement

The experimental work involved extraction of blood samples from healthy volunteers. In the present series of experiments, two of the co-authors (AB and EZ) were such volunteers from whom the blood is drawn. Approval for this was obtained from the University Ethics Committee, Manipal University, on 11 December 2008 (reference UEC/35/2008 and UEC/36/2008) and written, informed consent was obtained from the two co-authors whose blood was taken. All the experimental work reported in this paper relied only on blood samples taken from the co-authors, AB and EZ.

1 ml of whole blood was extracted from co-authors AB and EZ and was centrifuged at 3000 rpm for 5 minutes to remove the serum. The RBCs were diluted in Phosphate Buffered Saline (PBS) to get a suspension of living RBCs. About 10 µl of serum was again added to 1 ml of suspension to ensure that the RBCs remained intact for an extended period of time. For collecting while blood cells (WBC) same centrifugation procedure is followed. About 20 µl of serum near the buffy coat layer was pippetted out and diluted in 1 ml of PBS to obtain a solution of live WBCs.

### Apparatus

We first discuss a number of considerations that need to be addressed in the process of designing an apparatus that would find practical application in the biomedical sciences. On the trapping of biological matter, an important question relates to potential laser-induced damage within the trapping volume. Such potential hazards are expected to be wavelength and power dependent. For instance, photo-chemically induced alterations in the properties of trapped cells might include damage to DNA, alterations in cell metabolism and chemical effects like oxidation [28]. The tight spatial focusing that is inherent in optical tweezers inevitably leads to localized laser intensities that might easily exceed several MW cm^−2^, leading to localized thermal effects such as the formation of bubbles [29] and acoustic waves [30], leading to photomechanical effects that might prove to be deleterious to trapped biological matter. We refer to a recent, cogent discussion of such experimental considerations by Snook and coworkers [15] in the light of which we have designed and implemented the following experimental scheme.

**Figure 1:**
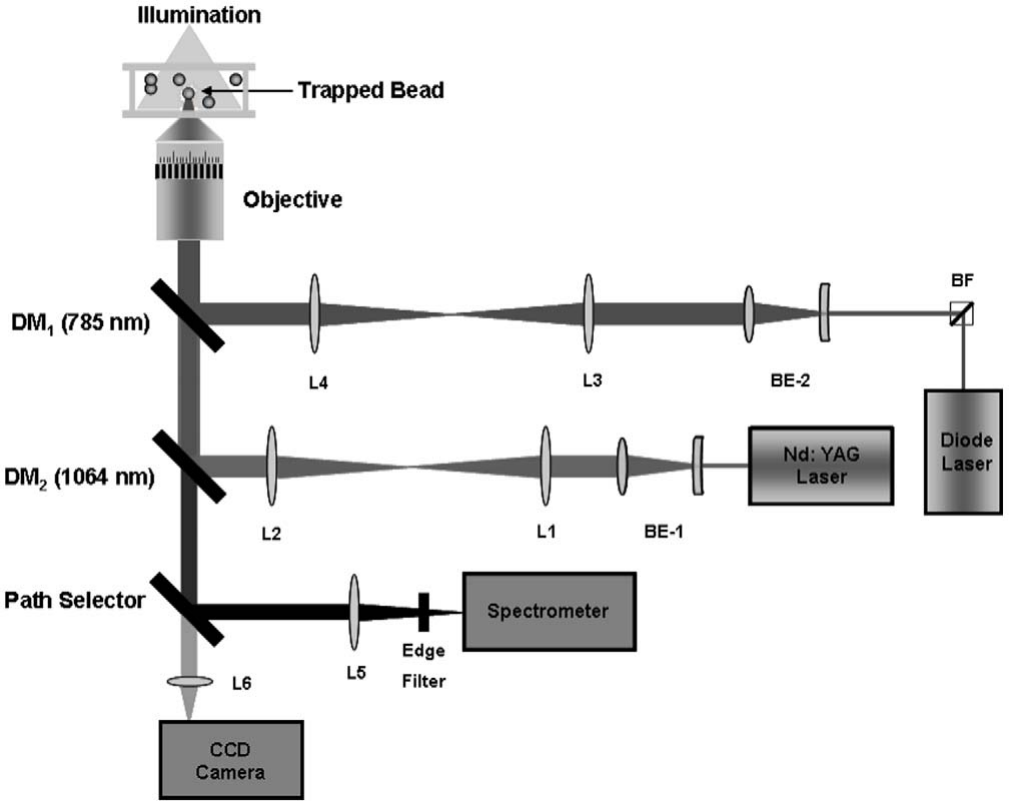
Schematic representation of our Raman Tweezers set-up. BE: beam expanders, L: lenses, DM: dichroic mirrors, BF: band pass filter used to “clean up” the spectrum of light from the diode laser used for Raman excitation (see text).

Figure 1 is a schematic depiction of the Raman Tweezers setup that we have designed and assembled in our laboratory. Our optical tweezers mainly comprises an inverted microscope (Nikon Eclipse Ti-U, Japan) with a high numerical aperture (1.3 NA), 100X oil immersion objective (Nikon, Plan Fluor) to produce a sharp focus to trap micro-particles suspended in solutions in a custom-made sample cell. The sample cell is made by attaching a microscope cover slip on to a metal plate with a 1 mm deep wedge of 0.8 cm width and 1 cm length. An Nd:YAG laser (Laser Quantum, UK) with an output beam of 1064 nm wavelength is used as the trapping laser. Measurements made on *E. coli* and CHO cells in the earlier reports have indicated that wavelengths in the close vicinity of 1 µm are expected to cause minimal laser-induced cell photodamage [31], [32]. In order to get a tightly focused, diffraction-limited spot at the sample, the back aperture of the objective has to be overfilled. We achieved this by expanding the beam from the Nd:YAG laser to nearly 9 mm diameter by means of a manual beam expander (BE1).

The laser beam was amenable to manipulation by a 1∶1 telescopic arrangement comprising two convex lenses of equal focal length, f, at a distance of 2f from each other as well as from the back focal plane of the microscope objective. A dichroic mirror (DM_2_) which has high reflectivity at 1064 nm wavelength directed the sufficiently expanded laser beam to the back aperture of our microscope objective. A CCD camera (Nikon DS-2MBW, Japan) was attached to one of the exit ports of the microscope, enabling visualization and recording of optically trapped micron-sized objects. Manipulation of the trapped objects was achieved simply by means of a controllable x–y translational stage.

Raman excitation of trapped particles was achieved in our apparatus by means of a 785 nm wavelength laser beam (Starbright Diode Laser, Torsana Laser Tech, Denmark) that was focused onto the sample by the same microscope objective. The choice of wavelength was dictated by the following considerations. The efficiency of Raman scattering exhibits a λ^−4^ wavelength dependence, indicating the desirability of using short wavelengths. However, as indicated by the work of Neuman and coworkers [31], short wavelengths result in increased propensity for laser-induced photodamage. The use of longer wavelengths also offers a potential advantage of reducing fluorescence effects that compete with the weak Raman signals that we anticipate in the type of work that we wish to undertake on biological samples. However, longer wavelengths also lead to inescapable problems that are related to the efficiency of CCD-based photon detectors that are readily available at present. As discussed by Snook and coworkers [15], the best available detection efficiency of ∼50% is obtained over the wavelength range 600–800 nm. The corresponding value around 1 µm wavelength is 10% while at 1064 nm it is almost zero. Our choice of 785 nm as the Raman excitation wavelength appears to be the optimum utilitarian compromise.

Might it be possible to utilize the 785 nm light for both trapping and excitation purposes? We have considered this possibility and, in some cases, the answer is in the affirmative. However, cognizance has to be taken of the fact that the stiffness of the optical trap strongly depends on the laser beam quality. The diode lasers presently available for Raman excitation have distinctly inferior beam quality to that obtained from a laser like the Nd:YAG laser that we utilize for trapping. Conseqently, use of the Raman laser for trapping results in considerably less efficient trapping. However, this can be tolerated under some circumstances. For instance, in some preliminary experiments we did use only the 785 nm laser beam for both trapping and excitation of RBCs, albeit less efficiently. This scheme certainly cannot be applied to WBCs whose refractive index (1.360±0.004) is somewhat lower than that of RBCs (1.399±0.006); hence a more efficient trap is required when work is to be conducted on WBCs.

**Figure 2:**
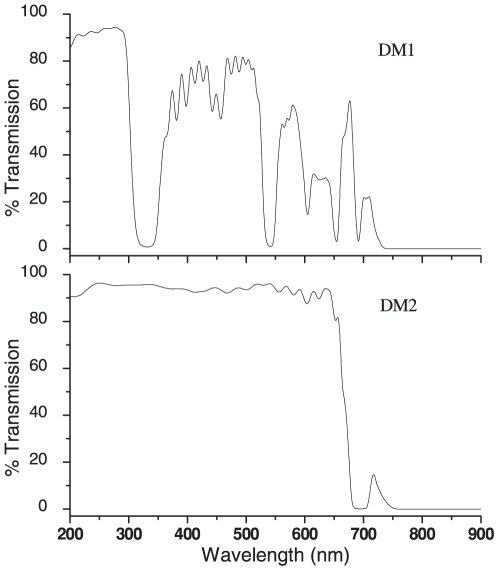
Characterization of the two dichroic mirrors used to transmit Raman signals to the spectrometer and visible light to the CCD camera. Transmission curves (% T) of dichroic mirrors (a) DM_1_ (785 nm) and (b) DM_2_ (1064 nm) over a wide range of wavelengths (200–900 nm).

In our experiments with the 785 nm laser we observed that the output beam contained an additional weak line around 790 nm which might interfere with the Raman signals. To circumvent this, we passed the laser beam through a holographic band pass filter BF (Kaiser Optics, USA) that effectively blocked the extraneous 790 nm line. The 785 nm beam was first expanded using a beam expander (BE2) so as to overfill the back aperture of the microscope objective and then reflected into the microscope by the 785 nm dichroic mirror (DM_1_). The combination of two dichroic mirrors was carefully chosen such that they transmitted Raman signals to the spectrometer and visible light to the CCD camera so as to facilitate visualization of trapped samples. Figure 2 shows the transmission curves that we measured for the two dichroic mirrors.

**Figure 3:**
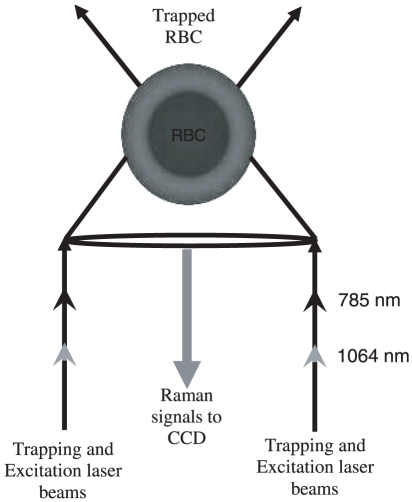
Dual-beam backscattering configuration employed in our Raman Tweezers set-up. Trapping laser (1064 nm) and probe laser (785 nm) beams are shown.

A 1∶1 telescopic arrangement allowed us to exercise proper control over the Raman probe beam. Using the telescopic arrangements both the laser foci was brought to the same point in the sample plane so as to enable simultaneous trapping and excitation. A backscattering configuration was used to collect the scattered signals from the trapped particle, as indicated in Fig. 3. The backscattered light was collected by the same objective and focused onto the spectrograph slit by an f/4 lens, which was coupled to one of the exit ports of our microscope. A 785 nm high pass edge filter (Razor Edge LP02-785RU-25, Semrock, USA) was used to exclude the intense Rayleigh scattering signal that inevitably accompanies the weak Raman signals. The Raman signals were dispersed using a Horiba Jobin Yvon *i*HR320 spectrograph with 0.6 nm resolution at 435 nm wavelength when using a 1200 grooves/mm grating blazed at 750 nm. Detection was by means of a liquid-nitrogen cooled, charge coupled detector (Symphony CCD-1024×256-OPEN-1LS) with 1024x256 pixels, operating at 140 K.

## Results and Discussion

**Figure 4:**
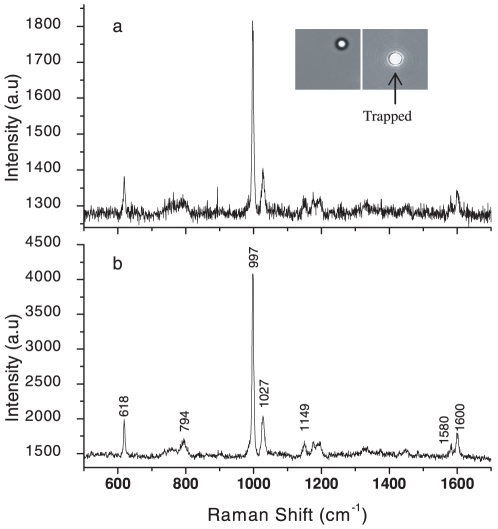
Alignment and calibration of the experimental set-up by using 785 nm laser light to ensure maximum signal-to-noise ratio in Raman spectra of 3 µm polystyrene beads. Raman spectra of a polystyrene bead trapped at ∼5 mW power and Raman excitation at ∼10 mW with (a) Acquisition time: 2 s (b) Acquisition time: 10 s. The insets show the image of the polystyrene bead before and after trapping.

Alignment and calibration of our set-up was accomplished using polystyrene beads of average diameter 3 µm (Sigma Aldrich, USA). A bead suspended in deionised water was trapped, typically using a trapping power of ∼5 mW, and the Raman spectrum was recorded by exciting it with 785 nm laser light. Raman spectrometer optics were aligned for maximum signal-to-noise ratio in measured spectra. Figure 4 shows typical Raman spectra of a trapped polystyrene bead with integration times of 2 s and 10 s. These spectra are consistent with those reported in the literature [4]. Typically, up to 20 spectra were recorded using 10 mW excitation power, and all of them are more or less identical to those shown in Fig. 4, with reproducibility on the wavelength axis being ±1 cm^−1^. Values of laser power used to trap cells and to record Raman spectra were measured by locating an integrating sphere just after the microscope objective in our set-up (Fig. 1). The spectral resolution of our system was determined to be ∼5.7 cm^−1^ (with the spectrometer slit width kept at 100 µm) by measuring the FWHM of the 997 cm^−1^ Raman band of the polystyrene bead spectrum. The inset to Fig. 4 shows an image of the trapped and untrapped polystyrene bead.

It is important to note here that laser power levels that were incident on the trapped cell were kept low enough to ensure that cell viability was likely to be maintained. In experiments whose results we report in the following, we typically made measurements on a single trapped cell for periods as long as ∼1 hour; in the course of this time we recorded Raman spectra every 5–10 minutes. The fact that recorded spectra was consistent during the entire stretch of the experiment helped allay concerns regarding cell viability. Moreover, other optical trap work conducted in our laboratory over several years [33]–[35] has provided evidence that optically-induced damage to RBCs and WBCs is unlikely to occur at incident laser power levels that are less than ∼30 mW in our system provided that the irradiated cells are kept in a physiologically relevant medium, which they were. The lowest possible incident power levels were utilized to obtain spectra presented in the following sections.

### Raman spectroscopy of trapped RBCs

**Figure 5:**
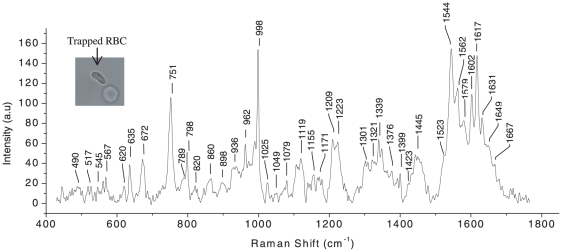
Raman spectrum of a trapped single RBC over the wavelength range 400–1750 cm^−1^. Laser power: ∼5 mW, acquisition time: 60 s, average of 5 accumulations. The inset shows the image of trapped and untrapped RBCs.

Raman spectra of optically trapped single RBCs and WBCs were measured with our apparatus using trapping and excitation power levels that are significantly less than earlier work [4]. The glass coverslip induced band in the region 1000–2000 cm^−1^ was overcome by using a fused silica coverslip which leaves only a peak at 798 cm^−1^ in the Raman spectrum. We first focus attention on spectra of trapped red blood cells (RBCs) of which typical spectrum is presented in Fig. 5.

A single living RBC was trapped, typically using ∼5 mW power, and Raman excited using typical power levels of ∼5 mW. Our recorded spectrum tends to cover a significantly larger region than that of earlier micro-Raman work [36]–[38] and several additional lines are observed in our measurements. Assignment of the vibrational bands observed in our spectra relied on the pioneering work on RBCs carried out by Wood and coworkers [36]–[38] and on Raman transitions in hemoglobin studies carried out by Hu and coworkers [39]. The complete assignment of spectra recorded by us in the present measurements is presented in Table 1.

**Table 1:** Observed Raman frequencies with corresponding band assignments for RBCs and whole blood-Serum.

Raman Band Positions in single RBC (in cm^−1^)		Raman Band Positions in whole blood (in cm^−1^)	Band Assignments	Raman Band Positions in single RBC (in cm^−1^)		Raman Band Positions in whole blood (in cm^−1^)	Band Assignments
Wood et al	Present work			Wood et al	Present work		
-----	473	478	ν_33_	-----	1138	-----	ν_43_
-----	490	-----	p: S-S str	1156	1155	1157	ν_44_
-----	517	517	p: S-S str	1174	1171	1170	ν_30_
-----	545	547	γ_21_, ν_25_, p: S-S str	1212	1209	1212	ν_5+_ ν_18_
567	567	570	ν(Fe^__^O_2_)	1226	1223	1224	ν_13_ or ν_42_
-----	589	584	ν_48_	1306	1301	1310	ν_21_
-----	620	621	γ_12, Phe:C-C twist_	1332	1321	-----	p:CH_2_ twist
-----	635	643	p:C–S str	1337	1339	1340	ν_41_
676	672	673	ν_7_	1371	1376	1376	ν_4_
754	751	752	ν_15_, γ_1_	1397	1399	1398	ν_20_
789	789	789	ν_6_	1430	1423	-----	ν_28_
827	820	824	γ_10_	1448	1445	1450	δ(CH_2_/CH_3_)
-----	860	855	γ_10_	1526	1523	1525	ν_38_
-----	898	898	p:C-C skeletal	1547	1544	1546	ν_11_
-----	936	927	ν_46_	1566	1562	1562	ν_2_
-----	962	971	p: Skeletal vibr	1582	1579	1583	ν_37_
974	989	989	γ(C_a_H = )	1604	1602	1603	ν_19_,ν (C = = C)_venyl_
993	998	997	ν_45_, Phe	1620	1617	1617	ν_10_, ν (C = = C)_venyl_
1031	1025	1026	δ( = C_b_H_2_)_asym_	1639	1631	1635	ν_10_
1047	1049	1050	ν(O = O), δ( = C_b_H_2_)_asym_	1653	1649	1649	Amide I
1074	1079	1066	δ( = C_b_H_2_)_asym_	----	1667		Amide I
1127	1119	1129	ν_5_				

Abbreviations: - ν & δ: In-plane modes, γ: Out -of- plane modes, sym: symmetric, asym: asymmetric, Str: stretching, p: protein, Phe: Phenylalanine.

The spectra contain a mixture of bands from the heme group and various protein components. The protein bands include the amide at 1649 cm^−1^ and the CH_2_/CH_3_ deformation modes primarily from amino acid side chains at 1445 cm^−1^. The Raman spectrum of RBC seems to be dominated by peaks corresponding to various vibrational modes of heme. The bands obtained here are well resolved and the apparatus that we have set up is clearly sensitive to even faint peaks. The richness of the spectrum brings to the fore the fact that Raman studies of live cells in suspension are more informative as far as molecular concentration and conformation are concerned than micro-Raman studies where the cells are chemically bound to a glass coverslip.

A comparison of our spectra with those reported by Wood and coworkers [36] shows that several new peaks are observed in our experiments; these are located at 1138 cm^−1^, 962 cm^−1^, 936 cm^−1^, 898 cm^−1^, 860 cm^−1^, 635 cm^−1^, 620 cm^−1^, 589 cm^−1^, 545 cm^−1^, 517 cm^−1^, 490 cm^−1^ and 473 cm^−1^. Most of these peaks are amenable to assignment on the basis of the conventional Raman measurements on hemoglobin conducted by Hu and coworkers [39]. The peaks 962 cm^−1^, 898 cm^−1^, 635 cm^−1^, 517 cm^−1^ and 490 cm^−1^ were not assigned in the studies of Hu and coworkers, though these lines may be contributed by the hemoglobin itself. We have assigned all these peaks in the present study using standard literature on vibrational spectroscopy of proteins [40] and we are able to conclude that the hitherto-unreported lines in RBC spectra obtained in the present study originate purely from hemoglobin molecules.

The presence of all these peaks in the present measurements on trapped RBCs may be a reflection of the relatively larger sample volume that is probed in our apparatus in comparison to that in the case of micro-Raman studies. Furthermore, the enhanced resolution in the present set of measurements may also be a contributory factor. In the micro-Raman study, the Raman probe beam is inevitably perpendicular to the plane of RBCs whereas in the case of trapped RBCs the orientation is parallel, and the probe beam passes through larger cell volume than is the case in micro-Raman measurements. In other words, the larger optical density in our experimental arrangement might contribute to higher signal sensitivity coupled with enhanced resolution that is achieved in the present experiments.

The concentration of hemoglobin in normal red blood cell is typically 32% to 36%; the domination of hemoglobin in IR spectra has been noted before and it has been postulated that this could be ascribed to excitonic coupling between aligned porphyrins in the highly aggregated heme environment of the RBC [31, and references therein]. Further work needs to be undertaken to gain a deeper insight.

**Figure 6:**
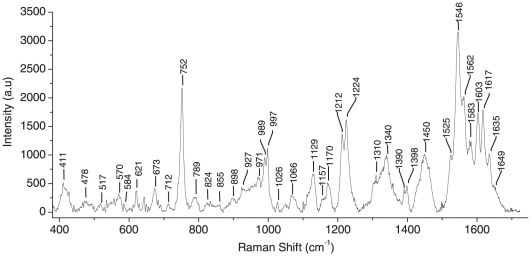
Raman spectrum of whole blood without serum over the range 350–1700 cm^−1^. Laser power: 20 mW, Acquisition time: 120 s, average of 5 accumulations.

A comparative analysis was also carried out by recording Raman spectra of blood samples after removal of serum as shown in Fig. 6. We measured a conventional Raman spectrum over the range 350 cm^−1^ to 1800 cm^−1^ and the peaks that we observed, along with their assignments, are presented in Table 2. Raman spectra of trapped RBCs closely resemble conventional Raman spectra of whole blood except that lines at 1423 cm^−1^, 1321 cm^−1^, 1138 cm^−1^ and 489 cm^−1^ are missing in whole blood spectra. These “missing lines” in whole blood spectra are most likely a consequence of the fact that the whole blood Raman spectrum is the ensemble average of many randomly oriented RBCs within the focused laser spot size (30 µm diameter in these measurements). However, further work needs to be undertaken to verify this conjecture of ours.

**Table 2:** Observed Raman frequencies with corresponding band assignments for WBC (Granulocytes).

Raman Band Positions in single Granulocyte (in cm^−1^)			Assignments	Raman Band Positions in single Granulocyte (in cm^−1^)			Assignments
Pupples et al.	Parker	present Work		Pupples et al.	parker	present Work	
	503	489	p: S-S str	-----	1100–1110	1104	p: C-N str
-----	519	514	p: S-S str	1126	1128	1120	p: C-N str
		543	p: S-S str	1145	1144	1142	DNA:RP(deoxyribose phosphate)
623	624	619	Phe:C-C twist	-----	1159	1156	p: C-C, C-N str
644	645	641	p: C-S str, Tyr: C-C twist	1175	1177	1179	Tyr,Phe,p:C-H bend
669	670	665	G,T,Tyr,G bk in RNA	1209	1210	1206	Tyr, Phe, A, T, p: Amide III
681	683	685	G	1232	1235	1234	p: Amide III
-----	710	699	C	1240	1248	1249	A, T, p:Amide III
729	730	723	A	1255	1260	1268	p: Amide III, C, A
750	752	754	T,Trp	1305	1304	1301	A, p: Amide III,Phospholipid:CH_2_
-----	774	772	C,T	1339	1340	1338	A, G, p: C–H def
783	787	782	C,T,U, Lip:O-P-O diester sym str	-----	1358	1359	Trp
833	830	829	O-P-O assym. str, Tyr	1375	1378	1372	T,A,G
853	850	850	Tyr	1400	1400	1389	U, A, IgG: COO^−^ sym str
-----	868	870	Ribose	1422	1423	1418	G,A
-----	880	877	Trp	1449	1449	1445	p:C–H_2_ def
898	900	900	DNA: bk, p: C-C skeletal	-----	1458	1453	p:(C–H3 def,C–H2 def),Rib:CH def
936	940	938	p: bk C-C str	1487	1491	1488	G,A
-----	963	962	p: skeletal vibration	-----	1552	1547	Trp, p: Amide II
-----	975	974	deoxyribose	1578	1580	1575	G,A
1004	1004	998	Phe: C-C skeletal	1606	1607	1604	Phe, Tyr, p: C = C
1032	1032	1026	Phe, p:C–N str	1617	1614	1618	Tyr, Trp, p: C = C
1048	1049	1040	Ribose Phosphate: CO str, p: C–N str	1659	1657	1660	p: Amide I
-----	1064	1059	Phospholipids: C-C str	1670	1672	1668	T(C = O),p:Amide I
1094	1083	1083	DNA:bk:O-P-O sym.str, p:C–N str				

Abbreviations: -def: deformation, bk: vibration of DNA backbone, U,C,T,A,G:ring breathing modes of the DNA/RNA bases, Tyr: Tyrosine, Trp: Tryptophan, Rib: ribose, NA: nucleic acids.

### Raman spectroscopy of trapped WBCs

White blood cells were also probed in the present series of experiments. Relatively little work has been reported [41], [42] on WBCs which are the mobile units of the immune system; they form about 1% of the blood of a healthy adult and serve to defend the body against infectious diseases and foreign materials [43]. WBCs consist of granulocytes, monocytes and lymphocytes. The granulocytes are produced in bone marrow and are characterized by abundant granules in the cytoplasm which represent packages of enzymes involved in the killing of ingested microbes and in digestion of phagocytosed material. Lymphocytes are produced in lymph nodes and spleen, are characterized by a large nuclear to cytoplasmic ratio, and have several roles in the immune system, including the production of antibodies and other substances that fight infection and diseases [43], [44].

**Figure 7:**
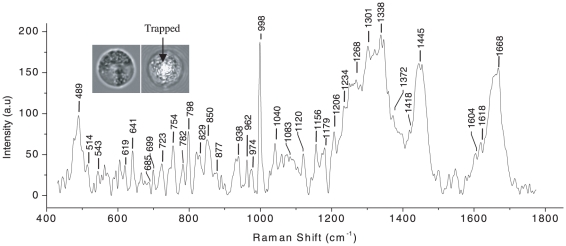
Raman spectrum of a trapped granulocyte over the wavelength range 400–1750 cm^−1^. Laser power: ∼10 mW, acquisition time: 120 s, average of 5 accumulations. The insets show the image of a granulocyte before and after trapping.

**Figure 8:**
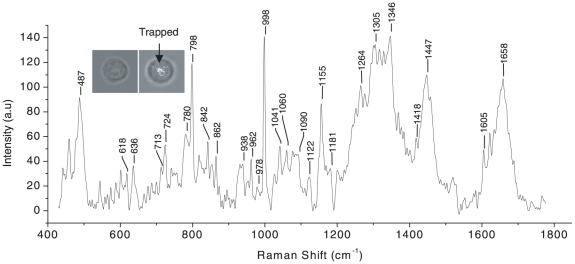
Raman spectrum of a trapped lymphocyte over the wavelength range 400–1750 cm^−1^. Laser power: ∼10 mW, acquisition time: 120 s, average of 5 accumulations. The insets show the image of a lymphocyte before and after trapping.

**Figure 9:**
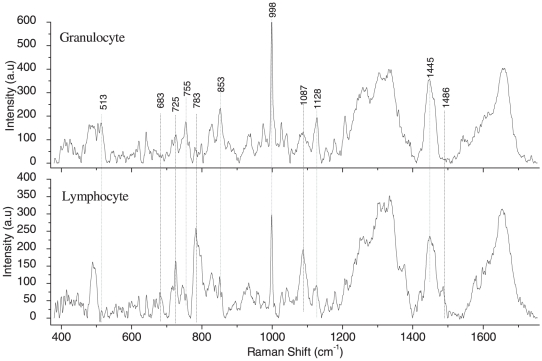
Raman spectra of trapped granulocyte and lymphocyte over the wavelength range 400 to 1750 cm^−1^. Laser power: ∼30 mW, acquisition time: 120 s, average of 5 accumulations.

Granulocyte and lymphocytes were trapped using typical power levels of ∼5 mW. Some un-trapped and trapped cells are shown in the inset to Figs. 7 and 8. Raman spectra of trapped granulocyte and lymphocyte measured using ∼10 mW power of Raman excitation are shown in Figs. 7 and 8 respectively. Raman spectra of trapped granulocyte and lymphocytes were also recorded at larger excitation power (30 mW) to test the quality of the spectra and stability of the cells and are shown in Fig. 9. The exposure of the WBCs to such power did not show any visible damage to the cells. The WBCs used here were collected from freshly drawn blood (within 10 minutes).

**Figure 10:**
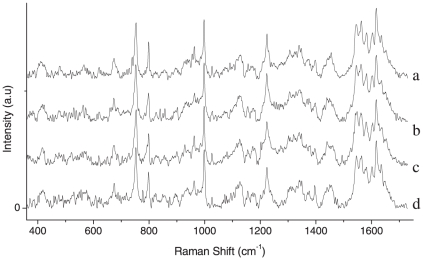
Raman spectra of trapped RBCs at a laser power of ∼5 mW. Acquisition time: 60 s, average of 5 accumulations. Spectra “a”–“d” are from different individual RBCs trapped at different times. Reproducibility of spectral features is determined to be better than ±1 cm^−1^ along the wavelength axis.

The vibrational spectra of lymphocyte and granulocytes are mainly dominated by the vibration due to proteins, lipids and nucleic acid bases, as expected from the Raman spectra of eukaryotic cells. The assignments of the Raman spectra of trapped granulocytes and lymphocytes are summarized in Tables 2 and 3, respectively. In comparing our data with the earlier work [41] on the granulocyte nucleus, we note that all previously reported Raman peaks are reproduced in our experiments. Additionally, we have discovered several new peaks that we assign on the basis of information available on standard proteins and nucleic acids [40]. Raman assignment of lymphocyte spectra was also made using available literature [40]. The Raman spectrum we obtained from trapped granulocyte consists of sharp peaks from proteins compared to the somewhat broader features in the spectra of trapped lymphocytes. This is likely a consequence of the dense granulated cytoplasm in the former. The peaks at 1445 cm^−1^, 1128 cm^−1^, 998 cm^−1^, 853 cm^−1^, 755 cm^−1^ and 513 cm^−1^ corresponding to proteins and amino acids are more intense in the granulocyte spectrum than the lymphocyte spectrum. On the other hand, the features at 1486 cm^−1^, 1087 cm^−1^, 783 cm^−1^, 725 cm^−1^ and 683 cm^−1^ corresponding to the vibrations of nucleic acid bases and DNA backbone stretching are more prominent in the lymphocyte spectrum. This may be because of the increased nucleus-to-cytoplasm ratio in lymphocytes. However, the Raman line at 975 cm^−1^ assigned to deoxyribose is very intense in granulocyte spectra (in comparison with lymphocyte spectra). The reason for these remains unclear at present. The above results seem to be more prominent in the Raman spectra recorded with 30 mW power (Fig. 9). Raman spectra of a few (different) RBCs are shown in Fig. 10. A comparison between these spectra shows a high level of reproducibility. Typically, reproducibility of spectral features depicted in Fig. 10 over the wavelength axis was better than ±1 cm^−1^, lending credence to the robustness of our methodology and the reliability of our spectroscopic information in Table 1, Table 2 and Table 3.

**Table 3:** Observed Raman frequencies with corresponding band assignments for WBC (Lymphocytes).

Raman Band Positions in single Lymphocyte (in cm^−1^)		Assignments	Raman Band Positions in single Lymphocyte (in cm^−1^)		Assignments
Parker	present Work		Parker	present Work	
503	487	p: S-S str	1064	1067	Phospholipid: C-C str
540	543	p: S-S str	1094	1090	DNA:bk:O-P-O sym str, p:C-N str
624	618	Phe: C-C twist	1100–1110	1110	p: C-N str
635	636	A,U,G,C	1128	1122	p: C-N str
670	677	G,T,Tyr,G bk in RNA	1159	1155	p: C-C, C-N str
710	713	C	1177	1181	Tyr,Phe,p:C–H bend
720	724	Phospholipid: Choline	1208	1200	Tyr, Phe, A, T, p: Amide III
752	756	T,Trp	1265	1264	p:Amide III;CH bend
787	780	C,T,U,Lip:O-P-O diester sym str	1304	1305	A, p: Amide III
835	842	DNA:RP(deoxyribose phosphate)	1320	1316	G,p:C–H2 twist
850	853	Tyr	1340	1346	A, G, p: C–H def
867	862	Ribose	1400	1401	U, A, IgG: COO- sym str
880	882	Trp	1423	1418	A,G
900	895	DNA: bk, p: C-C skeletal	1448	1447	Deoxyribose,p:C–H2 def
938	938	p: bk C-C str	1462	1458	Deoxyribose, p:(C–H3 def,C–H2 def)
963	962	p: skeletal vibration	1514	1519	A
975	978	Deoxyribose	1580	1583	G,A
1004	998	Phe: C-C skeletal	1607	1605	Phe, Tyr, p: C = C
1032	1026	Phe, p: C-N str	1650–1670	1658	p: Amide I
1049	1041	Ribose Phosphate: CO str, p: C-N str	1672	1674	T(C = O),p:Amide I
1055	1060	NA:C-O str, p:C-N str			

### Summary

A dual beam Raman Tweezers apparatus with superior performance has been assembled that involves a marriage of optical trapping methods with Raman spectroscopy. The utility of such a device for biomedical studies is illustrated by measuring Raman spectra of single red and white blood cells kept under physiological conditions. Raman spectra were recorded using sufficiently low power levels to ensure no photo-damage. Several hitherto-unreported Raman transitions were obtained in the present study and have been assigned. We believe that the work reported here reinforces the utility of and opens new vistas for Raman Tweezers to studies that aim to obtain molecular information from single living cells. The method can have wide applications in understanding the basic biochemistry and biophysics of RBC related diseases (Malaria, Sickle Cell Anemia, Thalassemia), cell-drug interactions, cell under stress, radiation induced cell damage, cell undergoing malignant conditions and many more. Work on cells under stress and malaria-infected RBCs is currenty underway in our laboratory.
